# Reduction of Displaced Acetabular Fracture with Central Hip Dislocation using Vector Traction: A Case Report

**DOI:** 10.5704/MOJ.2303.022

**Published:** 2023-03

**Authors:** RHY Wong, SM Lim, GMHJ Pang

**Affiliations:** Department of Orthopaedic Surgery, Hospital Queen Elizabeth, Kota Kinabalu, Malaysia

**Keywords:** vector traction, hip fracture-dislocations, hip joint reduction, hip protrusion, hip central dislocation

## Abstract

Fracture-dislocations of the hip is the result of high-energy trauma which necessitates urgent reduction. Closed reduction is usually attempted first and if failed, open reduction is indicated and may require more than one surgical approach. However, there is also the option of managing it with vector traction. This case report details the treatment of a middle-aged gentleman who sustained a left hip central dislocation which was gradually reduced with vector traction prior to surgery and in doing so, diminished the risk of him developing several potentially debilitating complications known to be associated with surgical fixation of such injuries.

## Introduction

Hip fracture-dislocations are typically caused by high-energy trauma, commonly from motor vehicle accidents^1^. These injuries require urgent reduction and surgery to restore joint stability and articular surface congruity^2^. If closed reduction fails, open reduction is indicated. Depending on the pattern of fracture/dislocation, more than one surgical approach may be needed, leading to prolonged surgical duration, more blood loss and other complications. Vector traction can be used for pre-operative reduction of acetabular fractures with concurrent hip dislocation to reduce the complexity of definitive surgery.

We report a case of T-type acetabular fracture with left hip central dislocation which was gradually reduced with vector traction prior to definitive surgery.

## Case Report

Our patient is a 32-year-old gentleman who sustained a left hip central dislocation with T-type acetabular fracture and left sacroiliac (SI) joint disruption from a motor vehicle accident. Closed manipulative reduction (CMR) was attempted multiple times in the casualty to no avail. CT scan showed a T-type acetabular fracture with medialisation of the left femoral head intrapelvis.

**Fig. 1:**
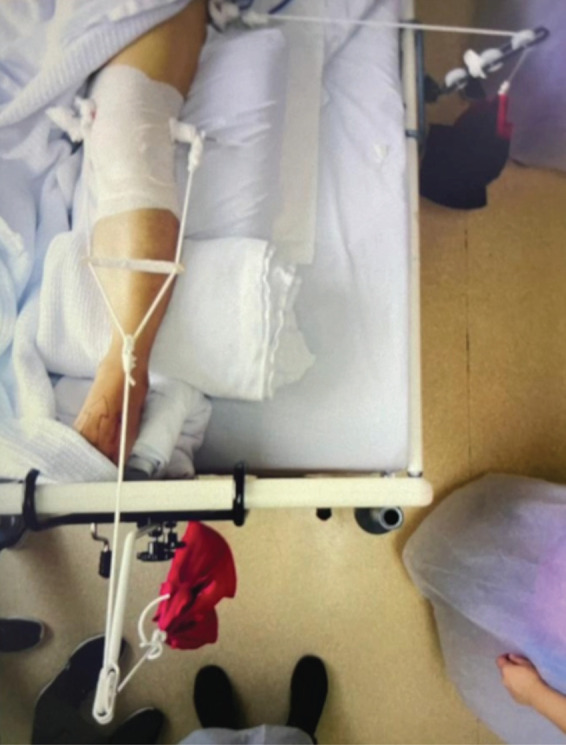
Clinical picture of vector traction showing one Schanz pin over the supracondylar region of the femur and another laterally inserted into the lesser trochanter.

Vector traction was used to gradually reduce his left hip fracture-dislocation prior to definitive fixation. Schanz pins were inserted into the supracondylar region and lesser trochanter of the femur, as shown in (Fig. 1). Traction was started at five kilograms (kg) each and increased by 5kg per day. Radiographs were repeated daily after each increment to evaluate the reduction progress.

**Fig. 2:**
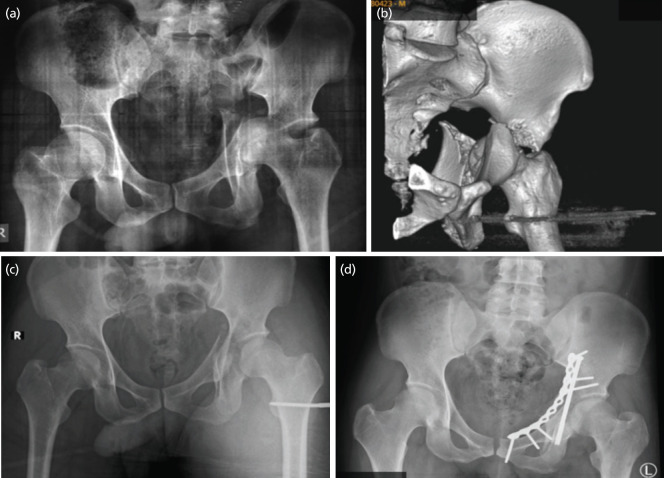
(a) Initial post-trauma pelvis AP radiograph showing fracture displacement and hip dislocation. (b) Pre-operative pelvis AP radiograph showing final reduction on vector traction 15kg. (c) Post-operative pelvis AP radiograph.

Initial post trauma pelvis radiograph and three-dimensional CT images are as shown in (Fig. 2a) and (Fig. 2b). After gradual increment up to 15kg, concentric reduction of the left hip was achieved as shown in (Fig. 2c). Iliopectineal and ilioischial lines were restored almost perfectly.

Anterior acetabular plating and posterior column screw fixation were performed one week post trauma via a single anterior incision using modified Stoppa approach. Intra-operatively, the SI joint was noted to be stable under fluoroscopy, hence fixation was not done.

Immediate post-operative radiographs showed a well-reduced hip as illustrated in (Fig. 2d). He was discharged well on day three post-surgery, with appointments at two weeks, one month, three months and six months. Throughout his follow-up, his wound healed well and radiographs showed that reduction was maintained. He started non-weight bearing crutches ambulation three months post-surgery. Unfortunately, due to the COVID-19 pandemic, he defaulted his follow-up.

## Discussion

Hip dislocations with or without acetabular fractures are usually due to high energy trauma. The femoral head may be forced medially through the floor of acetabulum due to a fall on the sides, a blow over the greater trochanter or axial loading of the femur^1^, resulting in a central hip dislocation, which is frequently associated with a transverse fracture of one or both acetabular columns^3^. Any hip dislocation must be reduced as soon as possible under anaesthesia and is usually achievable with CMR. Open reduction is indicated if closed reduction attempts fail.

Closed reduction via skeletal traction for central hip fracture-dislocations has been described in the literature. Tipton *et al* studied 38 patients with central fracture-dislocations, all treated non-operatively with different types of traction, and found that combined distal and lateral traction yielded best radiographical and clinical results^4^. The traction pins can be used during CMR to dis-impact the centrally dislocated femoral head before application of weights to maintain the achieved reduction.

Grubor *et al* described side and supracondylar extensions for temporary reduction and immobilisation of fractures until conditions for surgery are achieved. Traction with weights up to 20% body weight on femoral supracondylar traction, and up to 10% body weight on the side; resulted in a vector force in the direction of the femoral neck and tracts the head of femur from acetabulum via ligamentotaxis^5^.

Open reduction and internal fixation (ORIF) is often performed for accurate reduction and internal fixation for displaced acetabular fractures. Surgical approaches differ according to the type of associated fracture. A study conducted by Judet and Letournel in 1964 showed that a few cases of associated fractures of both anterior and posterior columns have been treated via anterior and posterior approaches in the same surgical setting^3^. Pantazopoulos *et al* described a combined anterior and posterior approach for T-type and two column fractures, in which satisfactory reduction was unattainable with a single posterior approach^2^. Despite being shown to have excellent results in treating acetabular fractures, ORIF is not without complications, which include surgical site infection leading to prolonged antibiotics, multiple debridement, and hip arthrodesis; sciatic nerve injury, heterotopic ossification, and avascular necrosis (AVN) of femoral head that necessitates total hip arthroplasty. Avoiding a second approach as in our case can possibly lead to reduced complications.

To our knowledge, vector traction as pre-operative reduction of hip fracture-dislocations has not been described in the literature and we would like to recommend it as a technique to reduce the complexity of definitive surgical fixation as well as risks of complications associated with ORIF.

However, it is important to noted that there may be risk of wound contamination and pin tract infection by the side pin inserted into the lesser trochanter if surgery is delayed and patient needs a posterior incision (eg. Kocher-Langenbeck approach) in addition to the anterior one.

We also recommend vector traction as a good means of temporary reduction if patient is unable to undergo surgery as soon as possible. The COVID-19 pandemic has caused a worldwide shortage of manpower and facilities in the healthcare sector, and in such cases, vector traction can provide temporary pain relief and comfort for patients whose surgeries had to be unfortunately delayed.

We were unable to assess the long-term complications associated with this method of treatment due to the loss of follow-up. However, we could be certain that the risks of short-term complications were minimised, the immediate post-operative outcome was good and the patient could be discharged early. Further comparative studies using vector traction versus a control group and comparing the operative difficulties, operative and fluoroscopic times, amount of blood loss and transfused, quality of fracture reduction, complications and outcome of surgery, should be carried out.
